# MicroRNAs orchestrating host and vector-borne protozoan interactions: bridging immune modulation and cross-species communication

**DOI:** 10.3389/fimmu.2025.1673237

**Published:** 2025-12-10

**Authors:** Jianyong Li, Feng Zhu, Jian Zhang

**Affiliations:** 1 Department of Pathogenic Biology, Army Medical University (Third Military Medical University), Chongqing, China; 2 Key Laboratory of Extreme Environmental Medicine, Ministry of Education of China, Chongqing, China

**Keywords:** host miRNA, vector-borne protozoan, immune regulation, cross-species communication, interaction

## Abstract

Vector-borne protozoan diseases, such as malaria, leishmaniasis, and trypanosomiasis, continue to impose a substantial burden on global health. The intricate molecular interplay between hosts and protozoan pathogens critically shapes disease progression and clinical outcomes, yet the regulatory mechanisms governing these interactions remain incompletely elucidated. Emerging evidence highlights microRNAs (miRNAs) as key epigenetic modulators of host-protozoan cross-talk. Infection with protozoans triggers dynamic alterations in host miRNA expression profiles, which subsequently fine-tune innate and adaptive immune responses through regulation of macrophage polarization, neutrophil activity, and T and B cell differentiation. Beyond their immunomodulatory roles, miRNAs can directly target protozoan genes to restrict pathogen replication, while protozoa, in turn, exploit host miRNA networks to evade immune surveillance and facilitate persistent infection. This bidirectional miRNA-mediated dialogue not only deepens our understanding of host-pathogen interactions but also unveils new avenues for clinical innovation. The distinctive expression patterns and functional versatility of miRNAs position them as promising candidates for diagnostic biomarkers, therapeutic targets, and next-generation intervention strategies. In this review, we synthesize the current knowledge on how miRNAs orchestrate the complex interplay between hosts and protozoa, and explore their translational potential in combating these neglected diseases.

## 1 Introduction

Vector-borne diseases account for over 17% of all infectious diseases, causing more than 700,000 deaths annually (1–3), which includes the Trypanosomatidae family (encompassing *Trypanosoma* and *Leishmania*) and Apicomplexan protozoa (exemplified by *Plasmodium*) (4, 5). Despite their taxonomic differences, from the perspective of host-parasite interactions, both groups employ sophisticated strategies to manipulate host immune responses, facilitating infection and disrupting immune homeostasis (6–8).The transmission strategies further highlight their differences: Trypanosomatidae rely on sand flies (for *Leishmania*) or tsetse flies (for African trypanosomiasis) and triatomine bugs (for Chagas disease) for transmission, whereas Apicomplexans such as *Plasmodium* are predominantly transmitted by *Anopheles* mosquitoes. Clinically, the diseases caused by these pathogens manifest differently. Trypanosomatidae infections have diverse presentations: *Leishmania* causes cutaneous and visceral leishmaniasis, while *Trypanosoma* species are responsible for human African trypanosomiasis (HAT) and Chagas disease, often progressing to chronic stages with neurological or cardiac complications, respectively. In contrast, Apicomplexan *Plasmodium* infections result in malaria, an acute febrile illness associated with high mortality rates in resource limited areas. Control efforts are further complicated by rising drug resistance, and the ongoing difficulties in vaccine development highlight the complexity of combating protozoan infections. To enhance diagnosis, treatment, and transmission prevention and control, it is important to fully elucidate the interactions among the mammalian (or vertebrate) host, the vector, and the protozoan. The main focus is currently on pairwise interactions, like those between the host and protozoan, but the regulatory mechanisms are not yet fully understood.

Most mammalian miRNAs, with a size varying between 20–22 nucleotides, regulate 1%–2% of mammalian genes. In this process, miRNAs bind specifically to mRNA and can degrade the transcript or hinder its translation into protein, while also upregulating the expression of target genes. These small RNA molecules are not only indispensable to biological events such as cell growth, cell specialization, cellular development, and even programmed cell death, but have also recently been found to be crucial in tuning the immune interactions between the host and pathogen (9, 10). It is important to establish how host miRNAs regulate the host immune response and interact with pathogens. Emerging evidence suggests that miRNAs act in many different capacities during host-pathogen interaction, primarily by targeting immune-related genes (11–13). In particular, in infections caused by protozoa, there are clear findings indicating that host miRNAs directly impact parasite survival and replication via alteration of innate immune response and adaptive immune response. For example, miRNAs impact macrophages, neutrophils, DCs and T, B cell function during *Plasmodium*, *Leishmania* and *Trypanosoma* infection (14–17).

MiRNAs also play an important role in interspecies genomic communication between the host and pathogen. For instance, the host miRNAs may control the gene expression of protozoa and thus influence the protozoans’ survival and reproductive success (18). In contrast, the protozoan can also hijack the host miRNAs (19). Cross-species associations elucidate critical dimensions of the dynamic interplay between protozoa and their hosts. In this review, we synthesize the current knowledge on how miRNAs orchestrate the complex interplay between hosts and protozoa. We also explore these interactions present promising avenues for advancing novel diagnostic and therapeutic strategies.

## 2 MiRNAs as regulators of gene expression

MiRNA biogenesis and functional mechanisms have been extensively studied (20). Small noncoding RNAs can arise from intergenic regions or from within genes, with approximately half of all known miRNAs being intragenic—derived principally from introns, and sometimes exons of genes—while the remainder are intergenic and are transcribed separately under the control of their own promoters (21, 22). Additionally, some miRNAs are transcribed in clusters comprising groups of miRNA genes that share sequence similarity; these miRNA clusters thus form functionally coordinated families that collectively operate within regulatory networks (23).

MiRNA biogenesis primarily occurs via canonical pathways, where RNA polymerase II first transcribes miRNA genes in the nucleus to form primary miRNAs (pri-miRNAs). These pri-miRNAs are then processed by the microprocessor complex, consisting of DiGeorge syndrome critical region 8 (DGCR8) and Drosha Ribonuclease III (DROSHA), into precursor miRNAs (pre-miRNAs) featuring a distinctive 2-nucleotide 3’ overhang (24, 25). Subsequently, the pre-miRNAs are transported to the cytoplasm via Exportin-5 and ras-related nuclear protein-GTP (RAN-GTP), where they undergo further cleavage by Dicer and TAR RNA-binding protein (TRBP) to generate mature miRNA duplexes. The functional strand of the duplex is then loaded into the RNA-induced silencing complex (RISC) alongside Argonaute (AGO) proteins, enabling sequence-specific binding to target mRNAs and subsequent post-transcriptional gene regulation (24, 25). This well-characterized pathway ensures precise control over gene expression through miRNA-mediated mechanisms.

Extensive research has shown that miRNAs play a crucial regulatory role in mammalian cells, potentially controlling approximately 30% of protein-coding genes through multiple mechanisms. These small non-coding RNAs can suppress protein production by interfering with key translation processes, including by blocking initiation factors, inhibiting ribosomal subunit binding with mRNA and blocking elongation during ongoing polypeptide chain synthesis on polysomes (26). MiRNAs also downregulate translation by recruiting cell machinery to degrade mRNA by deadenylating poly(A) tails and degrading mRNA in two directions (5’ and 3’), similar to translational repression (downregulated protein expression) (27–29). Crucially, these molecules do not have to remain within the cell; they can be actively loaded onto extracellular vesicles such as exosomes, or released into the body fluid where they act as intercellular messengers. The extracellular presence in combination with their specific expression during diseased states make miRNAs suitable candidates to serve as potential diagnostic markers for a wide array of conditions, particularly infectious diseases (30, 31).

## 3 Host miRNA regulates immune responses to vector-borne protozoan infection

MiRNAs are vital epigenetic regulators of immune gene expression, crucial for modulating host responses to protozoan infections. They fine-tune innate and adaptive immune pathways, affecting parasite control, immunopathology, and chronicity by targeting immune-related genes. Here, we explore the molecular mechanisms underlying miRNA-mediated immune regulation during these infections.

### 3.1 Host miRNA regulates the innate immune response

Innate immunity is responsible for recognizing pathogen-associated molecular patterns (PAMPs) via Pattern Recognition Receptors (PRRs), namely, C-type lectin receptors (CLRs), nucleotide oligomerization domain-like receptors (NLRs), retinoic acid-inducible gene I-like receptors (RLRs), AIM2-like receptors (ALRs), and Toll-like receptors (TLRs) (32–34). Specifically, TLR2 can recognize the alkylacylglycerol of *Trypanosoma cruzi* (35), and TLR2/TLR4 jointly recognize the glycoinositolphospholipids (GIPLs) and glycosylphosphatidylinositol (GPI) of the genus *Trypanosoma* (35). TLR9 recognizes the genomic DNA of the Trypanosoma species (35). TLR2 can recognize the lipophosphoglycan of *Leishmania* (35), and different species of *Leishmania* binding to TLR2 may trigger different functions. For example, in *Leishmania major* and *Leishmania donovani* infections, TLR2 may trigger host protective or non-protective immune responses; in *Leishmania mexicana* and *Leishmania infantum* infections, TLR2 mediates host protective responses; while in *Leishmania amazonensis* and *Leishmania braziliensis* infections, it may lead to disease exacerbation (36). SSC4D, belonging to the scavenger receptor cysteine-rich (SRCR) superfamily, can directly bind to *Leishmania* and *Trypanosoma*, promoting the phagocytic action of single cells (37). Recent studies suggest that miRNA plays an important regulatory role during protozoan infections by modulating PRRs and related signaling pathways. For example, miR-146 and miR-155 can negatively regulate the production of inflammatory cytokines by targeting CLR (e.g., Dectin-1) or downstream signaling adaptor proteins (e.g., Card9) (37), while other mechanisms are based on miRNA-TLR regulation (38, 39). It is speculated that protozoa influence TLR signal transduction by altering host miRNA expression; indeed, miR-223 can inhibit inflammasome assembly by directly targeting NLRP3 mRNA (40). Currently, most known miRNA regulatory mechanisms are derived from virus/bacteria models, and there is limited specific research on protozoa. Therefore, we will focus on the role of host miRNA regulation in innate immune cells during protozoan infections based on the limited studies available.

#### 3.1.1 Host miRNA regulation of macrophages and monocytes

**Figure 1:**
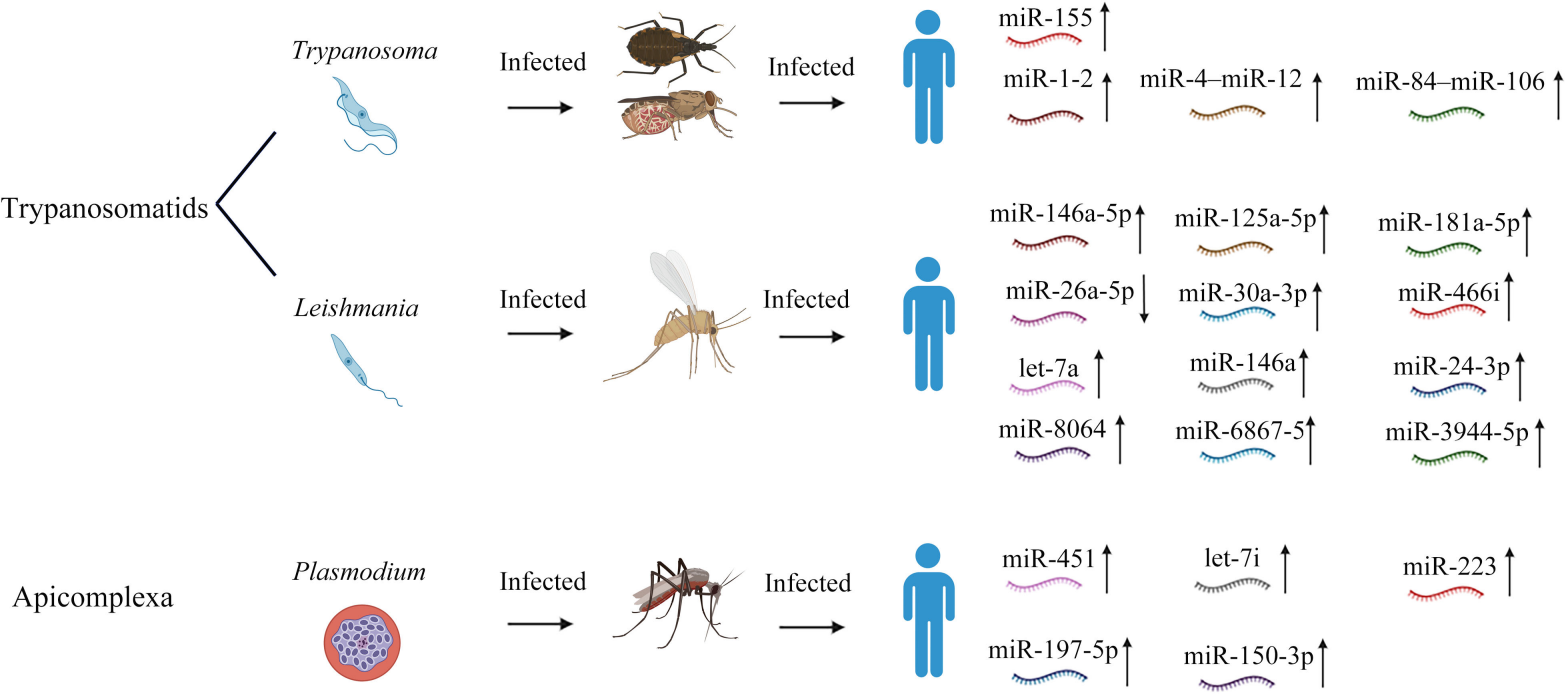
Schematic representation of the infection routes of three protozoa and the associated alterations in host miRNA expression. For each parasite, the diagram shows (i) transmission from the vector insect (tsetse fly, mosquito, or sand fly) to host, and (ii) the miRNAs that are significantly dysregulated in infected hosts. Upward arrows indicate miRNAs that are up–regulated, whereas downward arrows denote down–regulated miRNAs.

MiRNAs are crucial in modulating immune responses to protozoa like Plasmodium, Leishmania, and Trypanosoma by affecting monocyte and macrophage functions, particularly in macrophage polarization during these infections. In *L. donovani* infection, M2-associated miRNAs (e.g., miR-125a-5p, miR-181a-5p, miR-146a-5p) are upregulated, while the M1-linked miR-26a-5p is downregulated (41). Specifically, miR-146a-5p suppresses inducible nitric oxide synthase and enhances arginase 1 expression, supporting parasite survival (41). In contrast, *L. major* infection upregulates miR-146a, which promotes parasite clearance by targeting SMAD4 in the TGF-β pathway (42). Nimsarkar et al. demonstrated modulation of miR-146a isoforms during anti-*Leishmania* inflammatory responses (42), while Ganguly et al. showed that parasite-derived miR-146a is exported to uninfected cells to suppress inflammation and facilitate persistence (43). These species-specific differences may arise from distinct lipophosphoglycans interacting with varied TLR2 heterodimers, leading to divergent immune outcomes. Similarly, in malaria, Zhang et al. identified five *Plasmodium*-related miRNAs (miR-10a-5p, miR-10b-5p, miR-155-5p, miR-205-5p, and miR-21a-5p) that target Bcl-6 or SOCS-1, exacerbating liver injury by enhancing inflammation and M1 macrophage polarization (44). Hentzschel et al. corroborated these findings, observing strong IFN-γ pathway activation and elevated miR-155—particularly in liver macrophages—after injection with attenuated parasites (45) (**Figure 1**, **Table 1**). Although these miRNAs may aid in early parasite control through M1 responses, prolonged expression risks tissue fibrosis during recovery. Thus, therapeutic strategies targeting miRNAs must account for their timing-dependent and species-specific dual roles.

**Table 1:** Comprehensive summary of miRNA alterations and function in response to protozoan infections across host species.

Protozoan phylum/family	Protozoan species	Host species	miRNA name	miRNA function	Ref.
Trypanosomatids	*Trypanosoma cruzi*	Mouse	miR-155	Deficiency leads to reduced IFN-γ, TNF-α, CD8+ T cells, NK, and NKT cells, increased neutrophils and inflammatory monocytes, and enhanced host susceptibility.	(54)
	*Trypanosoma cruzi*	Mouse	let-7e-5p let-7f-5p miR-24-3p miR-21-5p	Acts as a central node in the miRNA-gene network, modulating macrophage response to *T. cruzi*.	(17)
	*Trypanosoma cruzi*	Mouse	miR-10a	Upregulated in thymic epithelial cells via TGF-β signaling, linked to infection-induced thymic atrophy.	(71)
	*Trypanosoma brucei*	Human/Mouse	miR-1–2 miR-4–miR-12 miR-84–miR-106	Predicted to modulate crucial virulence factors like variant surface glycoproteins during different parasite stages.	(74)
	*Leishmania donovani*	Human/Mouse	miR-146a-5p	Promotes M2 polarization, suppresses iNOS expression, and enhances Arg1 expression, facilitating parasite survival; in *L. major*, it targets SMAD4 to promote parasite clearance; and can be exported to uninfected cells to suppress inflammation.	(41–43)
	*Leishmania donovani*	Human/Mouse	miR-125a-5p miR-181a-5p	Associated with M2 macrophages, upregulated upon infection.	(41)
	*Leishmania donovani*	Human/Mouse	miR-26a-5p	Associated with M1 macrophages, significantly downregulated upon infection.	(41)
	*Leishmania donovani*	Human/Mouse	miR-30a-3p	Inhibits macrophage autophagy by targeting BECN1, promoting parasite persistence.	(49)
	*Leishmania donovani*	Human/Mouse	miR-466i	Activated via the NF-κB pathway, favoring early infection.	(19)
	*Leishmania donovani*	Human/Mouse	let-7a	Upregulated in infected DCs and macrophages, targets SOCS4, potentially modulating JAK-STAT signaling.	(59)
	*Leishmania major*	Human/Mouse	miR-146a	Targets SMAD4 in the TGF-β signaling pathway, promoting parasite clearance.	(42)
	*Leishmania major*	Human/Mouse	miR-24-3p	Upregulated in macrophages during early infection, modulates apoptosis.	(47)
	*Leishmania infantum*	Human	miR-8064 miR-6867-5p	Inhibits neutrophil phagocytosis by reducing CR3a expression.	(47)
	*Leishmania infantum*	Human	miR-3944-5p	Downregulates NCF4, suppressing oxidative burst and enhancing parasite survival.	(47)
Apicomplexa	*Plasmodium berghei*	Mouse	miR-155	Promotes M1 macrophage polarization, activates IFN-γ pathways; helps control parasites early but may cause tissue damage if prolonged.	(44, 45)
	*Plasmodium berghei*	Mouse	miR-21a-5p	Targets SOCS-1, potentially exacerbating liver inflammation and M1 polarization.	(44)
	*Plasmodium berghei*	Mouse	miR-10a-5p miR-10b-5p miR-205-5p	Targets Bcl-6 or SOCS-1, potentially amplifying inflammation and liver injury.	(44)
	*Plasmodium berghei*	Mouse	mmu-miR-16-5p	Targets multiple immune-related genes (AKT3, TLR1, CXCL10), potentially involved in neuroinflammatory signaling.	(60)
	*Plasmodium berghei*	Mouse	mmu-miR-491-5p	Targets genes like TLR9 and MYD88, affecting DC maturation.	(60)
	*Plasmodium falciparum*	Human	miR-451 let-7i miR-223	Suppresses PfEMP1 production, limiting parasite growth.	(73)
	*Plasmodium falciparum*	Human	miR-197-5p miR-150-3p	Targets parasitic apicortin, inhibiting parasite invasion and growth.	(18)

Host miRNAs regulate macrophage apoptosis and autophagy during *Leishmania* infection, altering cell survival. In *L. major* infection, miR-15a is downregulated while miR-155 is upregulated (46), and miR-24-3p increases during early infection (47). For *L. donovani*, miRNAs such as miR-8113, miR-763, and miR-3473f suppress apoptosis (48), and let-7a is elevated in both macrophages and dendritic cells (DCs), suggesting that anti-apoptotic miRNAs could serve as therapeutic targets. Additionally, early in *L. donovani* infection, upregulated miR-30a-3p inhibits autophagy by targeting BECN1, promoting parasite persistence (**Figure 1**, **Table 1**) (49).

Rego et al. identified let-7e-5p, let-7f-5p, miR-24-3p, miR-21-5p, and miR-155-5p as central nodes in the miRNA–gene network during *T. cruzi* infection, underscoring their key role in macrophage response modulation (17). *Trypanosoma brucei* and *T. cruzi* display distinct miRNA regulatory profiles: *T. brucei* upregulates miRNAs linked to the TNF-α/MIF axis, while *T. cruzi* perturbs the TGF-β/IL-10 pathway, with their differences attributable to differences in antigenic variation rates and parasitic lifestyles (intracellular vs. extracellular). *T. cruzi*’s intracellular adaptation involves mitochondrial TcFUT1 for metabolic and immune evasion (50), whereas *T. brucei* exhibits flexible extracellular enzyme regulation (51). *T. brucei* relies on RNA editing mechanisms, while *T. cruzi* may depend more on host nuclear pathways. Virulence factors such as cruzain in *T. cruzi* facilitate intracellular survival, while, in *T. brucei*, TbrCATL functions extracellularly (**Figure 1**, **Table 1**) (52).

In summary, host miRNAs affect immune responses by regulating the functions of macrophages and monocytes, albeit with varied mechanisms. *Leishmania* infection mainly upregulates miRNAs such as miR-146a, promoting M2 macrophage polarization and inhibiting apoptosis and autophagy, thereby benefiting parasite survival. *Plasmodium* infection drives M1 polarization through miRNAs such as miR-155, amplifying the inflammatory response, which helps control the parasite in the early stage but may lead to tissue injury in the later stage. *Trypanosoma* infection shows species differences, with intracellular *T. cruzi* disturbing the TGF-β/IL-10 pathway, while extracellular *T. brucei* upregulates TNF-α/MIF-related miRNAs, reflecting specific regulatory roles under different parasitic strategies.

#### 3.1.2 Host miRNA regulation of neutrophils

MiRNAs are crucial for neutrophil growth and function. Early in *Leishmania* infection, neutrophils perform specific roles such as forming extracellular traps and producing cytokines, possibly sheltering *Leishmania* before it infects macrophages (53). *Leishmania* can reduce neutrophil activity; however, some studies have emphasized the importance of neutrophil recruitment for host protection (53). Neutrophil responses vary among *Leishmania* species. Scaramele et al. found that increased levels of miR-8064 and miR-6867-5p can hinder neutrophil phagocytosis of parasites by reducing CR3a (Complement receptor 3, Integrin Subunit Alpha M) after the initial parasite uptake (47) (**Figure 1**). Once *Leishmania* is internalized, its LPG disrupts NADPH oxidase (NOX) complex formation by hindering CYBA/CYBB and NCF1/NCF2/NCF4 binding, reducing the oxidative burst and enhancing *Leishmania*’s resistance to subsequent immune attack (46). This study showed that the mRNA levels of four components of the NOX complex (CYBA, CYBB, NCF1, and NCF4) decreased after *L. infantum* infection. This downregulation, along with increased miR-3944-5p expression, which reduces NCF4 mRNA levels, may represent a survival mechanism induced by infection (**Figure 1**, **Table 1**) (47).

In Chagas disease, T. cruzi infection triggers a complex immune response, with neutrophils crucial for early defense. Research indicates that miR-155 is a key immune regulator. In miR-155-deficient (miR-155^-/-^) mouse models, the survival rate of mice significantly decreases after infection with *T. cruzi*, and the parasite load increases, indicating that the lack of miR-155 exacerbates the severity of the infection (54). Further immunological analysis reveals that miR-155 deficiency leads to reduced production of interferon-γ (IFN-γ) and tumor necrosis factor-α (TNF-α), both of which are crucial for controlling intracellular parasites like *T. cruzi* (54). At the same time, miR-155 deficiency also causes a reduction in CD8+ T cells, natural killer (NK) cells, and NK-T cells, while the accumulation of neutrophils and inflammatory monocytes increases, suggesting that miR-155 maintains immune balance by regulating various immune cell subsets, including neutrophils (54). The abnormal accumulation of neutrophils may be related to the dysregulation of inflammatory signaling pathways caused by the lack of miR-155 (54). For example, miR-155 typically limits excessive inflammatory responses by inhibiting specific target genes, but its deficiency may make neutrophils more prone to recruitment to the site of infection, thereby exacerbating tissue damage (54). In addition to miR-155, other miRNAs may also indirectly affect neutrophil responses in *T. cruzi* infection. For instance, in a general immune context, miR-223 has been reported to be involved in neutrophil differentiation and NET formation, while miR-146a influences neutrophil activation by regulating inflammatory signaling pathways (55, 56). However, the specific roles of these miRNAs in *T. cruzi* infection require further investigation. Current literature lacks clear experimental data on the direct interference of host miRNA with neutrophil function by *Plasmodium*. Although direct evidence is lacking, changes in host miRNA and inflammation mediators may indirectly affect neutrophil function. For example, IL-8 is an important neutrophil chemotactic factor, whose changes in expression during the infection process may alter neutrophil recruitment and activity (**Figure 1**, **Table 1**) (57).

Overall, the regulatory role of host miRNA on neutrophils shows significant differences. *Leishmania* parasites (e.g., *L. infantum*) upregulate miR-8064, miR-6867-5p, and miR-3944-5p to inhibit the phagocytic function of neutrophils (e.g., reducing CR3a expression) and oxidative burst (e.g., decreasing NOX complex components), thereby weakening their bactericidal ability and promoting parasite survival. In *T. cruzi* infection, miR-155 plays a key role, and its absence leads to abnormal accumulation and dysfunction of neutrophils, exacerbating the severity of infection. However, the roles of other miRNAs (e.g., miR-223 and miR-146a) remain unclear. Currently, there is a lack of direct evidence indicating that host miRNA directly regulates neutrophils in malaria infection, which may only indirectly affect their recruitment and activity through inflammatory factors (e.g., IL-8). Overall, the miRNA regulatory mechanism of *Leishmania* is the most clearly defined, *Trypanosoma* relies on the immune balancing role of miR-155, while research related to malaria is still limited.

#### 3.1.3 Host miRNA regulation of DCs

DCs contribute to linking the innate and adaptive immune systems by providing key cytokines and co-stimulatory molecules that activate T cells to initiate an immune response (58). Recent studies have demonstrated that miRNAs play important roles in the regulation of DCs during protozoan infections.

Next-generation sequencing has revealed distinct patterns of miRNA expression in human monocyte-derived dendritic cells (MDDCs) and macrophages (MDMPs) after infection with either *L. major* or *L. donovani* (both representing pathogenic strains responsible for different clinical manifestations) (59). Upon *L. donovani* infection, miR-511 is upregulated and interferes with the TLR4 signals necessary for an efficient immune response. Though Let-7a, which targets SOCS4—a JAK-STAT signaling inhibitor—is upregulated in *L. donovani*-infected cells, it is downregulated in *L. major*-infected cells. Moreover, targeting of the TGF-β signaling inhibitor SMAD by miR-21 has been found to exhibit an inverse correlation with SMAD7 expression in *L. donovani*-infected DCs. Conversely, miR-146b-5p has been found to be positively correlated with TRAF6 expression in *L. donovani*-infected DCs, indicating its involvement in TGF-β signaling feedback mechanisms (59). Furthermore, in *L. major* infections, PU.1 has been established to exhibit dual association with miR-155, acting as both a regulator and target, although no significant correlation between let-7a and SOCS4 has been detected in either infection type (59). These findings highlight the species-specific nature of miRNA signatures and corresponding transcript variations. In *Plasmodium berghei* ANKA infection, Aparecida et al. identified mmu-miR-16–5p and mmu-miR-491 as potential regulators of a substantial portion of differentially expressed gene (DEG) targets in splenic MDDCs (60). Additionally, on the basis of network construction and predictive analysis, the authors revealed that mmu-miR-16–5p potentially targets ten DEGs (AKT3, APP, CD40, CXCL10, HLA-DQA1, IFNGR2, IRAK2, MAPK9, PTGS2, and TLR1), all of which are upregulated and indirectly associated with the activation of neuroinflammatory signaling pathways (60). Comparatively, mmu-miR-491–5p was found to target six DEGs (TLR9, MYD88, ICAM1, HLA-DQB1, HLA-DMA, and FSCN1), all of which are also indirectly linked to DC maturation (**Figure 1**, **Table 1**) (60).

The direct research evidence of miRNA regulating DCs during *T. cruzi* infection in the host is relatively limited. However, numerous studies have reported the extensive role of miRNA in regulatory roles and cardiac pathological processes after infection, which may indirectly affect the function of DCs. Studies have shown that he expression of multiple miRNAs in the host cardiac tissue is significantly changed after *T. cruzi* infection, and these miRNAs are involved in regulating immune-related pathways such as the IFN-γ, TNF-α, and NF-κB signaling pathways (61). These pathways are closely related to the activation and function of DCs. Moreover, miR-146a is upregulated in the cardiac tissue, plasma, and extracellular vesicles of mice during the acute phase of infection and the chronic uncertain phase (62). MiR-146a is known to negatively regulate the NF-κB signaling pathway, which may affect the maturation of DCs and the production of inflammatory factors. Research has shown that the changes in expression of miR-145-5p and miR-146b-5p in infected cardiomyocytes are related to parasite load (63). These miRNAs may indirectly influence the antigen presentation process of DCs by regulating the host cell immune responses.

Overall, host miRNA regulates DCs in a species-specific manner. In *L. donovani* infection, miR-511 upregulates TLR4 signaling, let-7a targets SOCS4, and miR-21 inhibits SMAD7 to enhance TGF-β-driven DC tolerance, while miR-146b-5p is positively correlated with TRAF6. *L. major* infection involves bidirectional regulation of PU.1 and miR-155, while let-7a is downregulated. In *P. berghei* ANKA infection, mmu-miR-16-5p targets multiple upregulated genes (e.g., AKT3, CD40) associated with neuroinflammatory pathways, while downregulation of mmu-miR-491 may enhance antigen presentation and affect DC maturation. Existing literature lacks direct elucidation of the specific mechanisms by which miRNA regulates DCs in *T. cruzi* infection, with the majority of research focusing on the regulation of miRNA on cardiomyocytes and the overall immune response. Further experimental validation is needed to establish the direct association between miRNA and DC function. Overall, *Leishmania* tends to involve regulation of immune signaling pathways, while *Plasmodium* regulates inflammation and maturation processes.

### 3.2 MiRNA regulation of the adaptive immune response

Adaptive immune responses are primarily mediated by the activation and expansion of T and B cells, resulting in cytotoxic activity and antibody production against infections, respectively. MiRNAs are key regulators of adaptive immunity, influencing the development, activation, survival, and proliferation of these cells (64, 65).

#### 3.2.1 MiRNA regulation of T cell differentiation and activation

The involvement of miRNAs in the proliferation, differentiation, and maturation of CD4^+^ T cells during parasitic infections is well established. In the study of *Plasmodium* infection, miR-451, as an important regulatory molecule, shows significant differences between *in vitro* and *in vivo* models. In human sickle cell disease erythrocytes, the increased expression of miR-451 may limit the *in vitro* proliferation of *P. falciparum* by targeting the parasite’s translation process (58). However, *in vivo* studies have reached the opposite conclusion. In non-lethal malaria mouse models (e.g., those infected with the XNL strain of *Plasmodium yoelii*) miR-451 knockout (miR-451^−/−^) mice exhibited a faster parasite clearance rate, and this phenotype depended on the immune response of hematopoietic cells (66). Further mechanistic studies indicated that the protective phenotype is primarily driven by CD4+ T cells: CD4+ T cells in miR-451^−/−^ mice proliferated significantly more after infection, but their activation status was not significantly different from that of wild-type (WT) mice (66). This enhanced proliferation is partially achieved by relieving the inhibition on the target gene Myc, a key factor regulating cell cycle processes. The absence of miR-451 leads to upregulation of Myc expression, thereby promoting the clonal expansion of CD4+ T cells and changes in related gene expression, accelerating immune-mediated parasite clearance (66). The contradiction between *in vitro* and *in vivo* results may stem from the inherent differences in model systems: *in vitro* experiments focus on the direct effects of miR-451 on the parasite, while the *in vivo* environment involves complex interactions among multiple cell types and organ systems. Additionally, the expression level of miR-451 (e.g., increased or decreased) may trigger different biological effects; for instance, *in vivo*, its absence may exert protective effects by enhancing adaptive immune responses, while overexpression may directly affect parasite growth. These findings suggest that caution is needed when extrapolating *in vitro* miRNA research results to *in vivo* applications, as the role of miR-451 in overall host regulatory roles may extend far beyond its cell-autonomous effects.

Leishmaniasis outcomes are determined by the polarization of CD4^+^ T cells into Th1 or Th2 subsets (67). MiR-29a and miR-29b suppress Th1 differentiation by targeting T-bet, whereas miR-126 and miR-135 inhibit Th2 differentiation by targeting GATA3 (**Figure 1**, **Table 1**) (53). Additionally, miR-21 and miR-590-5b reduce Th1 responses by modulating IL-12 expression, while miR-98 and let-7a suppress Th2 by targeting IL-10 (68). Infected macrophages can modulate Th1/Th2 polarization, primarily by altering IL-12 signaling. For example, miR-155 knockout mice exhibit reduced IFN-γ production, indicating diminished Th1-associated responses. Naive CD4^+^ T cell differentiation is governed by Notch and JAK-STAT signaling, with lineage-specific transcription factors (TFs) directing Th1 or Th2 fates. Upregulated miRNAs targeting Notch3 reduce IFN-γ production, thereby promoting parasite survival. Several key miRNAs also modulate the JAK-STAT pathway and impact the IFN-γ response, though these mechanisms require further elucidation (69). Among the downregulated miRNAs, miR-340-5p enhances the differentiation of naive CD4^+^ T cells into the Th2 subtype by upregulating IL-4 expression and targeting IL-2 and IL-13 (70). Additional research is needed to clarify its contribution to Th2 polarization in *Leishmania* infections. Moreover, miR-93-3p and 486a-3p influence TFs such as STAT5 and STAT6, which in turn regulate downstream genes such as NLRP3 and *IL-4* (70). Differentially expressed miRNAs in CD4^+^ T cells also regulate PKC activity, cell division, MAPK cascades, SMAD binding, and protein dimerization processes (**Figure 1**, **Table 1**).

In *Trypanosoma* infections, B6 mice infected with *T. cruzi* show an increase in miR-10a expression within thymic epithelial cells—the cells that play a crucial role in the intrathymic differentiation of T cells. This upregulation of miR-10a is likely mediated by TGF-β signaling and appears to be linked to the thymic atrophy that develops as the infection progresses, as referenced previously (71).

#### 3.2.2 MiRNA regulation of B cell function

B cells that originate from primary lymphoid organs and mature in secondary lymphoid organs are vital for antibody-mediated immunity. Recently, various miRNAs have been found to be essential for different steps in B cell maturation and differentiation (72), among which miR-150, a ubiquitous miRNA in mature lymphocytes, blocks the expression of the transcription factor c-Myb, which is crucial for lymphocyte development and B-cell differentiation (72) (**Figure 1**). Although much effort has focused on the effects of *Plasmodium*, *Leishmania*, and *Trypanosoma* infections on B cell numbers and functionality, direct evidence for host B cells being influenced via miRNA regulation is still fairly limited.

## 4 MicroRNAs mediate cross-species communication between the host and protozoan

Recent advances have revealed miRNAs as pivotal mediators of this cross-species communication through reciprocal regulation of gene networks. Here, we focus on two critical mechanisms: how host-derived miRNAs target essential pathways for protozoan invasion and persistence, and how protozoans actively manipulate the host miRNA landscape to evade immune detection and establish infection. By integrating these perspectives, we illuminate the dynamic miRNA-mediated dialogue that shapes pathogenic relationships, offering novel insights into therapeutic interventions aimed at disrupting this delicate molecular balance.

### 4.1 Host miRNAs target key pathways for protozoan survival and invasion

**Figure 2:**
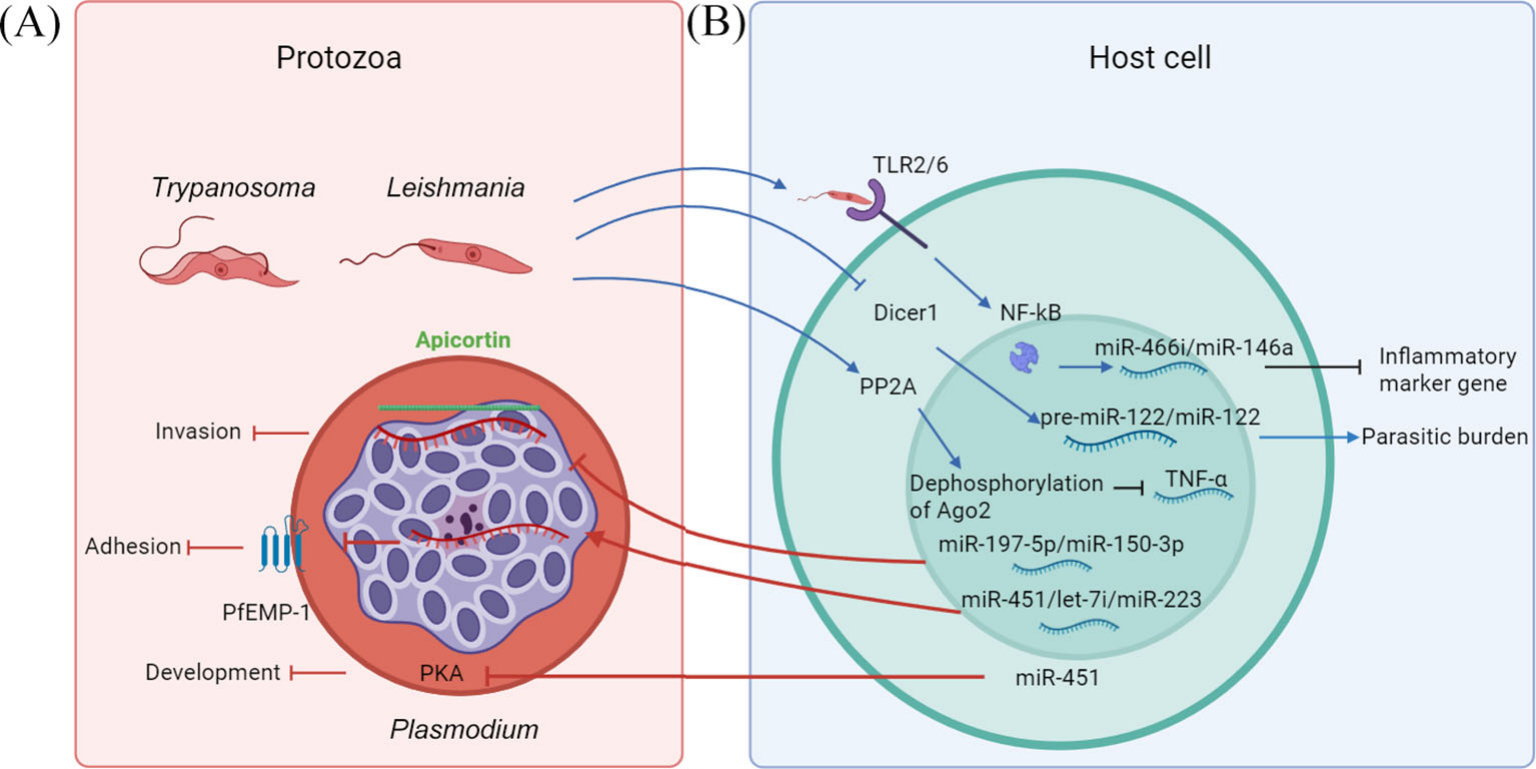
Schematic diagrams of miRNAs involved in host and protozoan interactions. The schematic is divided into two panels: **(A)** Protozoan (red background). Two representative protozoan morphologies are shown at the top left. The trypomastigote is described as the elongated form of *Trypanosoma*, characterized by its undulating membrane, while the promastigote is identified as the flagellated form of *Leishmania*. The central large cell is an erythrocyte containing an schizont, which is a stage of the parasite with multiple nuclei, within the parasitophorous vacuole. Host miR-197-5p and miR-150-3p could bind to the parasite’s apicortin and impair the process of parasite invasion. Host miR-451 and let-7i, along with miR-223 suppress the erythrocyte membrane protein-1(PfEMP1) production of the parasite. MiR-451 incorporation into the PKA-R gene resulted in reduced PKA regulation. **(B)** Host cell (blue background). Parasites interacted with the TLR2/TLR6 heterodimer to induce the transportation NF-κB into the cell nucleus; miR-466i and miR-146a expression was highly upregulated, causing downregulation of inflammatory marker gene expression. Protozoan have been shown to increase protein phosphatase 2A (PP2A) expression, PP2A-mediated dephosphorylation of AGO2 promotes AGO2 binding to miRNA, causing downregulation of TNF α production. Protozoan binds and cleaves host Dicer1, lowering the levels of pre-miR-122 and miR-122, inhibiting the miRNA maturation process, and altering host mRNA–miRNA interaction, hence facilitating the parasitic burden. Red arrows denote host miRNAs acting on parasite genes, such as apicortin/AMA1, PfEMP1 (adhesive protein), PKA (protein kinase A), thus affecting the process of protozoan invasion, adhesion, and development. Blue arrows extend from the parasite to the host cell, indicating parasite manipulation of host miRNAs.

Sequencing and bioinformatics analyses have revealed that the *P. falciparum* genome lacks essential RNAi machinery, including Dicer and AGO enzymes. Consequently, the parasite may rely on host-derived enzymes and miRNAs to regulate its gene expression. Recent studies show that erythrocyte-derived miRNAs, notably miR-197-5p and miR-150-3p, could bind to the parasite’s microtubule-stabilizing protein apicortin (18). When synthetic mimics of these miRNAs were introduced into erythrocytes, parasite growth and invasion were markedly impaired. Micronemal secretion was also reduced, especially in the case of miR-197-5p. Correspondingly, apicortin expression decreased in miRNA-loaded cells. The study further revealed that secretion of apical membrane antigen 1 (AMA1), a microneme protein critical for invasion and vaccine development, was also suppressed under miRNA treatment (18).This set a new stage for potential novel antimalarials that could be nucleotide-based against apicortin, while establishing miR-197-5p as an antimalarial therapy based on miRNA antagonists. Furthermore, elevated levels of miR-451 and let-7i in sickle cell erythrocytes, along with miR-223, were found to suppress *P. falciparum* erythrocyte membrane protein-1 production (73). The cyclic AMP pathway, crucial for parasite development, operates through cAMP-dependent protein kinase (PKA), with both its catalytic (PKA-C) and regulatory (PKA-R) subunits present in *P. falciparum* (73). Reducing PKA-R expression was found to be essential for parasite survival, whereas miR-451 incorporation into the PKA-R gene resulted in reduced PKA regulation and subsequent reduction of parasite growth (**Figure 2**) (73).

*T. brucei* harbors up to 881 predicted miRNAs that are arranged within genomic clusters encoding identical precursor molecules such as miR-1-2, miR-4–miR-12, and miR-84–miR-106. These are likely to be responsible for modulating crucial virulence factors at different stages of parasite development and growth including variant surface glycoproteins (74). In contrast, although *T. brucei* retains functional RNAi machinery—including AGO1—this system is absent in *T. cruzi*, *L. major*, and *L. donovani*.

### 4.2 Protozoan manipulation of the host miRNA Network

Protozoa possess the ability to disrupt host miRNA signaling pathways, thereby modulating the host’s immune responses and cellular functions. This interference enhances their infectivity and promotes an intracellular environment conducive to their survival.

#### 4.2.1 Protozoa affect host miRNA transcription

Similar to protein-coding genes, miRNA genes feature CpG islands, TATA box sequences, and transcription start elements and are subject to histone modifications. These characteristics indicate that miRNA promoters are regulated by TFs, enhancers, silencing elements, and chromatin modifications (75). Numerous TFs positively or negatively modulate miRNA expression in development- and tissue-specific manners (76). Studies have revealed that infection with *L. donovani* can alter miRNA expression profiles in host macrophages. In particular, the host transcription factor c-Myc may bind to miRNA gene promoters, influencing their deacetylation, leading to inhibition of miRNA biosynthesis and promoting parasite survival. Notably, inhibition of c-Myc activity has been found to be associated with reduced survival of the flagellated protozoa (77).

The surface expression of N-acetylgalactosamine is present in antimony-resistant *L. donovani* strains and not in antimony-sensitive strains. This glycoconjugate allows *L. donovani* parasites to engage in selective interaction with the TLR2/TLR6 heterodimer to induce the transportation of p50/c-Rel subunits of NF-κB into the cell nucleus (19). In macrophages infected with *L. donovani*, miR-466i expression was highly upregulated, accompanied by several differentially expressed miRNAs. Analysis through promoter prediction software indicated a potential regulatory region situated approximately 5 kb upstream of the miR-466i gene region that contained three NF-κB binding motifs (19). When NF-κB antagonists were applied, *L. donovani*-induced miR-466i expression levels were substantially attenuated (19). Promoter deletion experiments demonstrated that the complete activation of miR-466i in *L. donovani*-infected cells depends on all three NF-κB binding sites, indicating an indispensable role for NF-κB during this regulation (19) (**Figure 2**). A more recent study determined that NF-κB p65 mediated miRNA induction in epithelial cells in the *Cryptosporidium parvum* infected host (78), whereas, in *L. donovani*-infected macrophages, the increased expression of miR-466i was associated with nuclear translocation of NF-κB subunits p50/c-Rel, but not p65. Additionally, as mentioned above, *P. falciparum* infection was reported to upregulate miR-146a levels (79) (**Figure 2**), whose promoter region contains NF-κB-binding sites, confirming its NF-κB dependency (80).

#### 4.2.2 Protozoa affect the processing of host miRNA

The regulation of miRNA processing is a key determinant of miRNA expression profiles. This regulation involves enzymes such as Dicer and Drosha, their associated double-stranded RNA-binding protein partners, and mechanisms like miRNA editing and modulation of miRNA functionality. Both antimony-resistant and antimony-sensitive *L. donovani* strains have been shown to increase protein phosphatase 2A (PP2A) expression and decrease Hu-antigen R (HuR) expression, albeit to varying degrees (81). PP2A enhances miRNA function, whereas HuR acts antagonistically, diminishing miRNA function and thereby influencing cytokine responses of the host. PP2A-mediated dephosphorylation of AGO2 during *Leishmania* infection promotes AGO2 binding to miRNA, causing downregulation of inflammatory marker gene expression in infected macrophages (81). Over-expression of HuR induces robust pro-inflammatory response by inhibiting *L. donovani* through blocking PP2A activity (81). Some parasite virulence factors affect host miRNA activities and consequently regulate host mRNA expression via direct targeting of host miRNA processes. Moreover, in *L. donovani*, gp63 glycoprotein binds and cleaves host Dicer1, lowering the levels of pre-miR-122 and miR-122, inhibiting miRNA maturation process, altering host mRNA–miRNA interaction, hence facilitating parasitic burden enhancement in mouse liver (82). Additionally, *L. amazonensis* arginase, the enzyme converting L-arginine to ornithine and urea, can affect macrophage miRNA expression in the case of infection (83) (**Figure 2**).

## 5 Translational potential

### 5.1 Diagnostic and prognostic biomarkers

**Figure 3:**
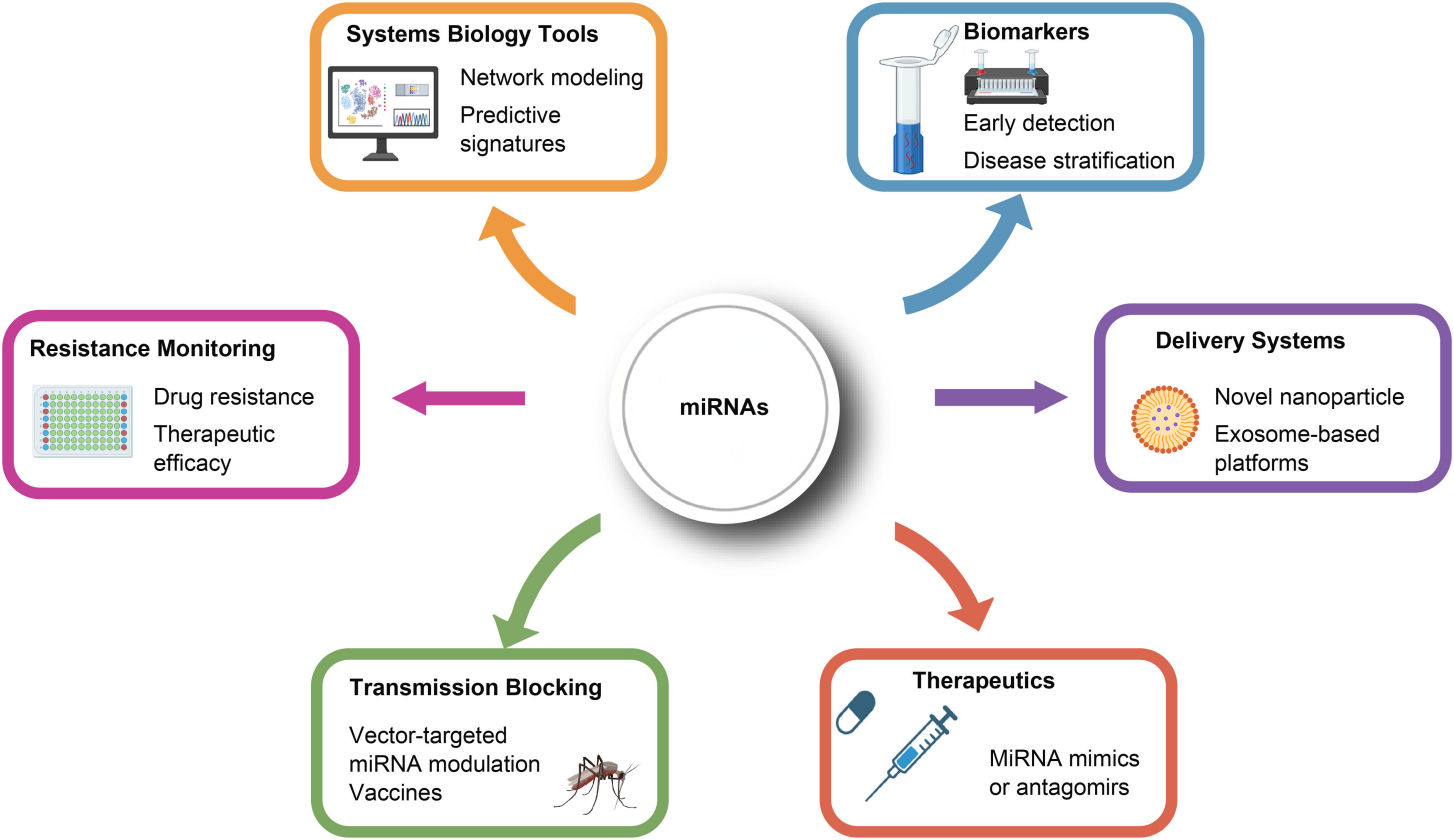
Translational applications of microRNAs in vector-borne protozoan diseases. This illustration summarizes the promising translational avenues enabled by miRNA research in protozoan infections. Diagnostic/Prognostic Biomarkers – miRNA signatures serve as non-invasive biomarkers for early detection, disease stratification, and treatment monitoring. Therapeutics – miRNA mimics or antagomirs are developed to modulate host immune responses or directly inhibit pathogen survival. Drug Resistance Monitoring – Dynamic miRNA profiles provide insights into emerging drug resistance and therapeutic efficacy. Advanced Delivery Systems – Novel nanoparticle and exosome-based platforms enable tissue-specific miRNA delivery with reduced off-target effects. Transmission-Blocking Strategies – miRNA-based interventions target vector competence or parasite development within vectors to reduce transmission. Systems Biology – miRNAs facilitate integrative network modeling and predictive multi-omics analyses to reveal disease mechanisms.

In recent years, growing evidence has highlighted the regulatory roles of miRNAs during protozoan infections, offering promising avenues for real-world diagnostic and prognostic applications. For instance, in visceral leishmaniasis, plasma levels of miR-223-3p, miR-191-5p, and miR-1285-5p are significantly elevated in patients with active disease compared to healthy controls or individuals with post-kala-azar dermal leishmaniasis, suggesting their utility as non-invasive biomarkers for detecting active infection (84). To strengthen the translational potential of these findings, future studies could define standardized validation pipelines—such as multi-center cohort studies with well-characterized patient samples—and compare miRNA profiles across different disease stages and endemic regions. Similarly, in canine visceral leishmaniasis, increased expression of miR-21-5p and miR-146a-5p correlates with parasite load in peripheral blood mononuclear cells, while elevated extracellular vesicle concentrations further underscore the diagnostic relevance of miRNA signatures (85). Future work in this area would benefit from incorporating defined animal models (e.g., experimentally infected dogs or murine models) and developing portable detection platforms—such as paper-based assays or microfluidic devices—coupled with rigorous analytical validation. For African sleeping sickness, computational analyses suggest that tsetse fly-derived miRNAs (e.g., miR-15a-5p) may modulate parasite transmission via nerve growth factor signaling pathways, offering a theoretical foundation for stage-specific diagnostics (**Figure 3**) (86, 87). To translate these predictions into applicable tools, experimental validation using relevant biological samples—such as tsetse fly salivary glands or patient serum—should be integrated with proteomic or transcriptomic data. Establishing well-annotated sample cohorts from diverse epidemiological settings will help verify biomarker specificity and sensitivity, ultimately supporting the development of field-deployable diagnostic kits.

### 5.2 Development of treatment strategies

In terms of therapeutic strategies, the regulatory role of host miRNAs appears promising. For example, miR-548d-3p is upregulated in human macrophages infected with *Leishmania* parasites and inhibits parasite proliferation through the MCP-1/CCL2 and nitric oxide pathways, suggesting that it could serve as a target for therapies (88). To enhance practical relevance, future studies could employ specific models such as human macrophage cell lines (e.g., THP-1 cells) or animal models like murine infection systems to validate these effects, with sample cohorts including primary human macrophages from diverse donors to assess variability. Validation pipelines might incorporate functional assays (e.g., NO production measurements) and miRNA manipulation using mimics or inhibitors to confirm mechanistic roles. Additionally, bioinformatics predictions indicate that miRNAs like miR-135 and miR-126 may regulate the Th1/Th2 immune balance by inhibiting the GATA-3 transcription factor, offering novel avenues for immune therapy design (68, 89). Computational approaches, such as integrating multiple miRNA functional similarity networks, could refine these predictions and identify candidate miRNAs for experimental validation in immune cell cultures or *in vivo* models. For African sleeping sickness, the discovery of parasite-specific miRNAs (e.g., those targeting RPS27A and GAPDH) opens pathways for antiparasitic drug development. Future work should focus on using parasite culture models and sample cohorts from infected hosts to verify miRNA functions, coupled with validation through target degradation assays or electrochemical biosensors for rapid detection (**Figure 3**) (87).

### 5.3 Drug resistance monitoring and reversal

Research has demonstrated that specific miRNA expression profiles (miRNA signatures) are closely associated with chemotherapy resistance in diseases like cancer (90, 91), and this concept can be extended to protozoan infections. By monitoring the “miRNA characteristic profile” in patients’ serum or plasma, it is possible to rapidly assess the sensitivity of isolates to first-line drugs (e.g., antimonials and artemisinin derivatives), enabling personalized therapy and avoiding ineffective treatments. To improve applicability, future directions should include defined sample cohorts—such as longitudinal collections from patients with documented drug resistance—and validation pipelines using electrochemical biosensors for high-sensitivity miRNA detection. Moreover, resistance mechanisms often involve dysfunction in host immune pathways (92); for example, in antimonial-resistant leishmaniasis, macrophages exhibit dysregulated immunosuppressive pathways, and the downregulation of miRNAs like miR-146a or miR-21 can restore nitric oxide (NO) production, potentially reversing drug resistance. Experimental details could involve *in vitro* models using macrophage cell lines infected with resistant parasites, treated with miRNA-based regulators (e.g., antagomirs or mimics), and validated through quantitative PCR and functional assays to measure NO output and parasite viability. Integrating these approaches with computational networks for miRNA-disease association prediction could further optimize monitoring strategies and enhance the translational potential of miRNA-based interventions (**Figure 3**).

### 5.4 Advanced delivery systems and engineered exosomes

The clinical translation of miRNA therapeutics relies on sophisticated delivery platforms that ensure tissue-specific targeting and minimize off-target effects. For instance, lipid nanoparticles and polymeric nanocarriers functionalized with cell-specific ligands—such as mannose for macrophage targeting—can be employed to deliver miRNA modulators precisely to infected cells (93). In parallel, engineered exosomes have gained attention as natural, biocompatible carriers with intrinsic homing abilities. To strengthen experimental relevance, future studies could focus on developing exosomes modified with targeting peptides (e.g., cyclic RGD for endothelial targeting) and loading them with therapeutic miRNAs using electroporation or transfection methods. These engineered exosomes should be evaluated in established murine infection models, with biodistribution and targeting efficiency assessed via *in vivo* imaging or qPCR. Additionally, incorporating multi-dose regimens and toxicity profiling in relevant animal cohorts will help clarify translational potential. Such systematic validation will not only enhance accumulation in target sites such as the spleen and liver but also help reduce systemic toxicity, thereby improving therapeutic outcomes in preclinical settings (**Figure 3**) (94).

## 6 Discussion and future challenges

In recent years, omics research, such as transcriptomics and genomics, has revealed that infections from protozoan infections are able to regulate the expression of host miRNAs, which in turn orchestrate host–parasite interplay. Host miRNAs regulate immune responses in protozoan infections through distinct mechanisms involving macrophages, neutrophils, and DCs. *Leishmania* miRNAs promote M2 polarization and inhibit autophagy in macrophages, suppress neutrophil functions, and show species-specific effects in DCs (41–43), while *Plasmodium* miR-155 drives M1 polarization and inflammation (44), and *Trypanosoma* species infections exhibit species-specific regulatory differences in macrophages and neutrophils, with varying research depth (17, 50–52).

Moreover, miRNA regulates adaptive immune responses through differential mechanisms. In *Plasmodium* infection, there is a contradiction regarding the role of miR-451 *in vivo* and *in vitro* (58, 66). In *Leishmania* infection, the focus is on the regulation of CD4^+^ T cell polarization, where multiple miRNAs form a fine network (53, 67, 68). In *Trypanosoma (T. cruzi*) infection, miR-10a is upregulated in thymic epithelial cells by TGF-β signaling, which is associated with infection-related thymic atrophy, but its specific role in T cell function remains unclear (71). Regarding B cell regulation, direct evidence from the three parasitic infections is relatively limited; it is known that miR-150 plays a key role in B cell maturation by targeting c-Myb, but its specific regulatory mechanisms in infection still need further exploration (72).

MiRNAs also significantly influence cross-species interactions between hosts and protozoan parasites, which represents a remarkable evolutionary adaptation that significantly influences infection outcomes. For instance, host-derived miRNAs can directly target critical pathways in protozoan pathogens. Conversely, protozoan pathogens have developed sophisticated strategies to manipulate host miRNA networks. Moreover, future research should focus more on the interactions among protozoa, vectors, and hosts, in addition to studying their individual relationships. In natural transmission, vectors introduce both pathogens and bioactive saliva during blood-feeding, while also ingesting host factors that modulate vector competence. For instance, host blood components (e.g., TGF-β and insulin) affect malaria transmission (95, 96), and *Aedes aegypti* venom allergen-1 enhances dengue and *Zika* infectivity by triggering autophagy in monocytes (97). Similarly, sand fly saliva potentiates Leishmania infection by dampening local immunity (98, 99), though host immunity to saliva—such as against SP15—can confer protection via cellular responses (100, 101). Triatomine saliva impairs DC function and cytokine signaling, aiding immune evasion (102), and in silico prediction that triatomine miRNAs from salivary glands potentially target human blood-expressed genes, suggesting that they can modulate the host’s gene expression associated with immune and inflammatory responses at the bite site, influencing the establishment of infection by *T. cruzi* after vector blood feeding on the host (103). Notably, *Anopheles coluzzii* saliva contains miRNAs homologous to human miRNAs, potentially regulating host immune and inflammatory genes (104). Functional studies confirm that host-derived miR-150-5p agomir enhances mosquito flaviviral infection, whereas its antagomir curtails transmission (105). These findings emphasize the importance of tripartite interaction studies for controlling protozoan infections and designing strategies to block transmission (**Figure 3**).

To facilitate the translation of miRNA research into clinical applications, future studies should focus on evaluating the potential of miRNAs as biomarkers and developing them as therapeutic targets. However, challenges remain with the practicality of this research. Technical standardization is the primary obstacle, as differences in sample sources (e.g., plasma compared to peripheral blood mononuclear cells), RNA extraction methods, and detection platforms (e.g., NanoString versus qPCR) across studies lead to reduced data reproducibility (106). At the epidemiological level, in regions where malaria, leishmaniasis, and sleeping sickness co-occur, pathogen-specific miRNA signals (e.g., miR-451a in malaria) can easily be confused with general inflammatory responses; this necessitates validation of the specificity of multi-pathogen miRNA combinations in multicenter studies (107). In terms of technical application, the current detection methods lack sensitivity for low-abundance miRNAs and can be expensive, hindering the development of portable devices. Although nanoparticle tracking technology has confirmed the diagnostic value of the extracellular vesicle concentration in canine models, extending this technology to resource-limited areas requires new technology at the very least (108). Moreover, biologically, the differences in how miRNAs work across species mean that any results should be interpreted with caution (106, 109).

**Figure 4:**
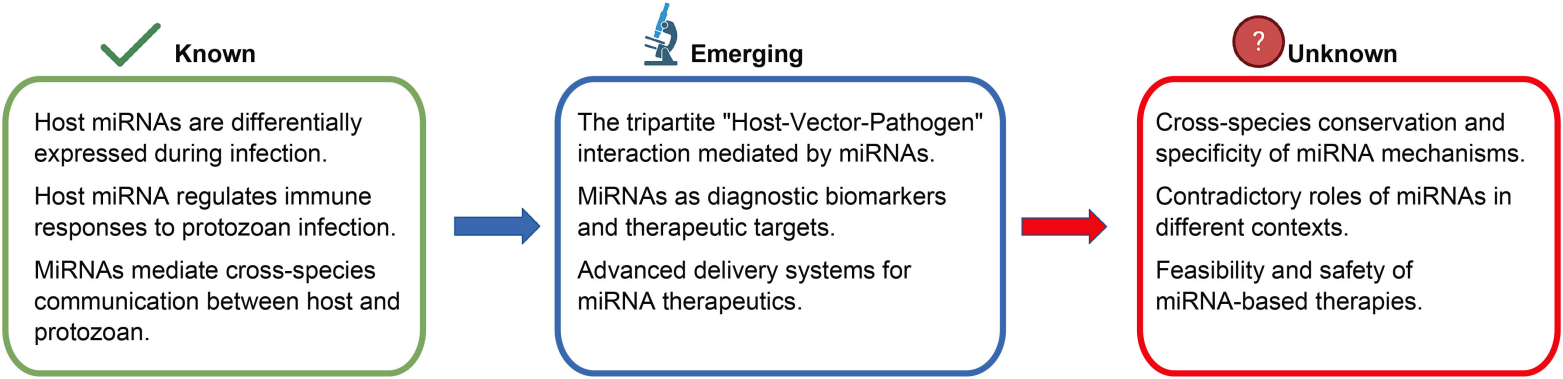
Categorization of knowledge on miRNA functions in host–protozoan interactions. Diagram presenting a three–panel classification of the current understanding regarding miRNA roles in host–protozoan interactions, progressing from established knowledge to emerging research and unresolved questions. Known (left panel, green checkmark): Summarizes the well–characterized functions of host miRNAs, including differential expression during infection, their ability to regulate the immune response and orchestrate cross-species communication between the host and protozoan. Emerging (middle panel, microscope icon): Highlights active research areas, such as the tripartite “Host–Vector–Pathogen” interaction mediated by miRNAs, the use of miRNAs as diagnostic biomarkers and therapeutic targets, and advances in delivery systems for miRNA–based therapies. Unknown (right panel, question mark): Identifies key open questions, including the cross–species conservation and specificity of miRNA mechanisms, the contradictory roles of miRNAs in different contexts, and the feasibility and safety of miRNA–based therapies. Blue and red arrows indicate the progression from established knowledge to emerging research and unresolved challenges, respectively.

Therefore, future research should focus on comparative miRNA profiling across vectors (e.g., mosquitoes, sandflies, triatomines) to identify species-specific signatures influencing immune responses, coupled with functional validation using CRISPR/Cas9 or antagomirs to resolve the contradictory roles of key miRNAs such as miR-451 in *Plasmodium* infections. Systems biology approaches, such as integrated network modeling of host–pathogen miRNA interactions, will help to elucidate regulatory hubs in immune pathways, while cross-species evolutionary assays are essential to confirm the conservation of miRNAs derived from different species in transmission and inform targeted anti-parasitic strategies (**Figure 4**). In summary, this review supports the emerging view that miRNAs serve as key mediators in host–protozoan communication, bridging immune modulation with cross-species regulatory dynamics. A deeper exploration of these mechanisms will enhance our understanding of host–pathogen interactions and offer innovative strategies for the prevention, treatment and blocking transmission of diseases caused by protozoa.
